# Substrate Utilization and Cycling Performance Following Palatinose™ Ingestion: A Randomized, Double-Blind, Controlled Trial

**DOI:** 10.3390/nu8070390

**Published:** 2016-06-23

**Authors:** Daniel König, Denise Zdzieblik, Anja Holz, Stephan Theis, Albert Gollhofer

**Affiliations:** 1 Section for Nutrition and Sports, Department of Sports and Sports Science, University of Freiburg, Schwarzwaldstrasse 175, Freiburg 79117, Germany; Denise.Zdzieblik@sport.uni-freiburg.de (D.Z.); AG@sport.uni-freiburg.de (A.G.); 2 BENEO-Institute, Wormserstrasse 11, Obrigheim 67283, Germany; Anja.Holz@beneo.com (A.H.); Stephan.Theis@beneo.com (S.T.)

**Keywords:** isomaltulose, athletic performance, fat oxidation, glycemic index, endurance

## Abstract

(1) Objective: To compare the effects of isomaltulose (Palatinose™, PSE) vs. maltodextrin (MDX) ingestion on substrate utilization during endurance exercise and subsequent time trial performance; (2) Methods: 20 male athletes performed two experimental trials with ingestion of either 75 g PSE or MDX 45 min before the start of exercise. The exercise protocol consisted of 90 min cycling (60% VO_2_max) followed by a time trial; (3) Results: Time trial finishing time (−2.7%, 90% CI: ±3.0%, 89% likely beneficial; *p* = 0.147) and power output during the final 5 min (+4.6%, 90% CI: ±4.0%, 93% likely beneficial; *p* = 0.053) were improved with PSE compared with MDX. The blood glucose profile differed between trials (*p* = 0.013) with PSE resulting in lower glycemia during rest (95%–99% likelihood) and higher blood glucose concentrations during exercise (63%–86% likelihood). In comparison to MDX, fat oxidation was higher (88%–99% likelihood; *p* = 0.005) and carbohydrate oxidation was lower following PSE intake (85%–96% likelihood; *p* = 0.002). (4) Conclusion: PSE maintained a more stable blood glucose profile and higher fat oxidation during exercise which resulted in improved cycling performance compared with MDX. These results could be explained by the slower availability and the low-glycemic properties of Palatinose™ allowing a greater reliance on fat oxidation and sparing of glycogen during the initial endurance exercise.

## 1. Introduction

Carbohydrates and fats are the most important energy sources during exercise [1,2,3,4]. It is well established that both a high carbohydrate diet before exercise as well as carbohydrate (CHO) ingestion during prolonged endurance exercise significantly improve endurance performance. However, for endurance athletes, there are several situations in training and competition in which high carbohydrate oxidation rates may not be desirable. These situations comprise phases of basic training in which fat metabolism should be improved or segments in endurance competitions (e.g., road cycling) in which the intensity is in the aerobic range and carbohydrate stores could be spared.

The human body has a distinct metabolic flexibility to switch between fat and carbohydrate utilization [3,5]. A major regulatory factor for substrate utilization is the presence or relative preponderance of one macronutrient over the other. Another important possibility of modulating substrate utilization consists in the modulation of the glycemic index (GI) of foods in the diet.

In recent years, the role of CHO in sports nutrition has been studied with respect to the GI. The GI provides a method of classifying foods based on their postprandial blood glucose response compared to a reference (i.e., white bread or glucose). At a given quantity, foods with a low GI result in lower postprandial blood glucose and generally also in a lower insulin response compared with high GI foods [6]. Insulin is the strongest hormone in suppressing fat oxidation. It has been shown that the GI of a meal has a significant effect on the postprandial fuel metabolism, both under resting conditions and during exercise [7,8]. Most investigations have found that the consumption of a low glycemic meal prior to physical exercise increased fat oxidation during endurance exercise compared with a higher glycemic meal [9,10,11,12,13]. Therefore, there is a rationale to consider the GI in the athlete’s diet as well as in consumed CHO before and during exercise since increased fat oxidation could promote endurance stamina and glycogen sparing in liver and skeletal muscles [14,15].

Commercial sports drinks most often contain high GI carbohydrates like for instance maltodextrin where the glucose monomers are linked by rapidly digestible α-1,4-glycosidic bonds. Isomaltulose (Palatinose™) is a disaccharide with glucose and fructose linked by an α-1,6-glycosidic bond. The low GI of Palatinose™ of 32 [16] results from the slow hydrolysis of the α-1,6-glycosidic bond by the sucrose-isomaltase complex situated on the brush border membrane of the small intestinal cells [17]. Therefore, the rate of absorption of Palatinose™ is rather slow. Nevertheless, after hydrolysis, glucose and fructose are efficiently taken up in the small intestine, and it has been shown that Palatinose™ is a fully digestible carbohydrate [18].

The aim of the present investigation was to analyze the influence of isomaltulose (Palatinose™) vs. maltodextrin ingestion on substrate utilization during endurance exercise and subsequent time trial performance in trained cyclists. The hypothesis was that isomaltulose ingestion before exercise would favor fat oxidation during the initial endurance exercise leading to glycogen sparing in the muscle and liver. The spared glycogen would then be available for improved performance during the time trial.

## 2. Materials and Methods

### 2.1. Subjects

Twenty male athletes participated in this study (age 29 ± 3 years; weight 75.6 ± 1.1 kg; height 183 ± 1.1 cm; VO_2_max 61.3 ± 1 mL/kg/min). Subjects were eligible if they were healthy experienced endurance cyclists (VO_2_max > 55 mL/kg/min) having participated in previous tests on cycling ergometers with time trial events. All subjects completed a comprehensive medical examination and routine blood testing. Written informed consent was given by all subjects and the study protocol was approved by the ethical committee of the University of Freiburg. The exercise tests was performed at the Cycling Lab (Radlabor) Freiburg.

### 2.2. Design

The study employed a randomized, double-blind, controlled cross-over design. Each subject attended the laboratory on 4 occasions (2 preliminary sessions followed by 2 experimental trials). During the first preliminary session, the individual VO_2_max was determined using a ramp test (cycling 3 min at 100 W and 3 min at 150 W, then increasing 10 W/10 s until exhaustion). During the second preliminary session, subjects performed a pre-test with a commercial sports beverage for better familiarization with the exercise protocol (conditions were identical to that used in the experimental trials described later). The implementation of such a familiarization has been shown to increase the reliability of subsequent tests [19].

During the remaining two test sessions, subjects performed the exercise protocol following ingestion of 750 mL of a beverage containing either 75 g isomaltulose (Palatinose™, PSE) or maltodextrin (MDX) (10% *w*/*v*). Both drinks were of comparable sweetness and identical in terms of taste and appearance. After an overnight fast, the different carbohydrate drinks were ingested in randomized order 45 min prior to the start of the exercise protocol. Randomization as well as the preparation of the different carbohydrate drinks was carried out by a person not actively involved in the conduct of the study. The exercise protocol commenced with 90 min of endurance exercise (cycling at the individual 60% VO_2_max as determined during the first preliminary session). After the endurance exercise, a time trial test followed immediately; the test was finished when a workload of 6.5 kJ/kg bodyweight was achieved. Subjects were instructed to finish the test as fast as possible. All exercise protocols were performed on the same day of the week (i.e., at least 1 week apart) and at the same time of the day in order to minimize effects of circadian variation. All testing was done using the same cycle ergometer.

In the evenings before the tests, subjects ingested a standardized supper at 7 pm consisting of a commercially available pasta meal (670 kcal, 21.5 g protein, 61.5 g carbohydrate, 38.5 g fat). Afterwards, they were only allowed to consume water or unsweetened tea until the beginning of the test sessions. During their participation in the study, subjects maintained a constant duration and intensity of training.

### 2.3. Parameters

The primary outcome parameter in this study was the time needed to finish the time trial. Secondary outcome parameters comprised the power output during the final 5 min of the time trial as well as the profiles of several physiological parameters including blood glucose and lactate concentrations, changes in substrate oxidation, and heart rate.

Capillary blood samples were drawn at −45 min (i.e., before ingestion of the drinks), −30, −15 and 0 min (i.e., immediately before the start of exercise) as well as after 15, 30, 45, 60, 75, and 90 min of endurance exercise and upon completion of the time trial. Blood glucose and lactate concentrations were determined enzymatically with ESAT 6660 (Medingen, Germany). Mean oxygen uptake (VO_2_) and carbon dioxide production (VCO_2_) were determined over 3-min intervals at −45, −30, −15, 0, 15, 30, 45, 60, 75, and 90 min using ZAN 600 CPET (nSpire Health Care, Oberthulba, Germany). The ratio of carbon dioxide production to oxygen consumption (respiratory quotient, RQ) was calculated and energy expenditure, carbohydrate and fat oxidation were determined according the equation of Weir [20]. Heart rate was recorded throughout the protocol (Polar Electro GmbH, Buettelborn, Germany).

### 2.4. Statistical Methods

Data were analyzed using the probabilistic magnitude-based inference approach as recommended for studies in sports medicine and exercise sciences. A published spreadsheet [21], designed to examine post-only crossover trials, was used to determine the mechanistic and clinical significance between conditions based on guidelines by Hopkins [22]. Analysis was done using Microsoft Excel version 2010 (Microsoft, Redmond, WA, USA).

Physiological outcomes were described by mechanistic inferences. The magnitude of the effect was tested for substantiveness against the standardized (Cohen) change of 0.2 times the between-athlete standard deviation for the reference condition (i.e., the maltodextrin trial). Interpretation of the magnitude of the effect was based on Cohen’s effect size (ES) scores of standardized differences and classified according to the following modified system: trivial (0–0.2), small (0.2–0.6), moderate (0.6–1.2), large (1.2–2.0), and very large (>2.0) [22].

Performance data were described by clinical inferences. The threshold for a substantial change was given by 0.3 times the typical within-athlete variability (coefficient of variation) to test for a small effect; moderate and large performance effects were described by 0.9 and 1.6 of the typical within-athlete variation [22]. The coefficients of variation for finishing time and power output following familiarization in a simulated cycling time trial of a comparable distance as in the current study have been determined previously and were reported as 1.5% and 3.6%, respectively [19].

An effect was unclear if the confidence interval (CI) overlapped both the upper and lower thresholds for substantiveness. Otherwise, the likelihood of a substantial increase or decrease was classified as follows: almost certainly not (<0.5%), very unlikely (1%–5%), unlikely (5%–25%), possibly (25%–75%), likely (75%–95%), very likely (95%–99.5%), and almost certainly (>99.5%). For mechanistic inferences, the threshold chances for substantial magnitudes were set at 5%. For clinical inferences, the threshold chances for harmful (“non-beneficial”) and beneficial effects were set at 0.5% and 25%, whereas an effect was declared beneficial if the odds ratio of beneficial/non-beneficial was >66 [22].

Unless otherwise stated, data are reported as raw means ± SD. Mean differences between trials are presented as the percentage change with the associated 90% CI. All metabolic and performance data were log-transformed prior to analysis to reduce non-uniformity of error and to express outcomes as percent [22].

Besides the probabilistic magnitude-based inferential analysis, statistical *p*-values for the comparisons between the MDX and PSE trial for all physiological and performance related parameters are also reported. A repeated measures analysis of variance (ANOVA) was used to identify significant effects attributable to time, trial, or both. Mauchly’s test was consulted and, if the assumption of sphericity was violated, the Greenhouse-Geisser correction was applied. If a significant time × trial interaction existed, simple main effects of trial were examined. A *p*-value of < 0.05 was considered statistically significant. Statistical analysis was done using SPSS Statistics Version 21 (SPSS Inc., Chicago, IL, USA).

## 3. Results

All twenty subjects completed the study and were included in the final analysis. Due to missing spirometric data from three subjects, results for fat and CHO oxidation were evaluated for *n* = 17.

**Figure 1:**
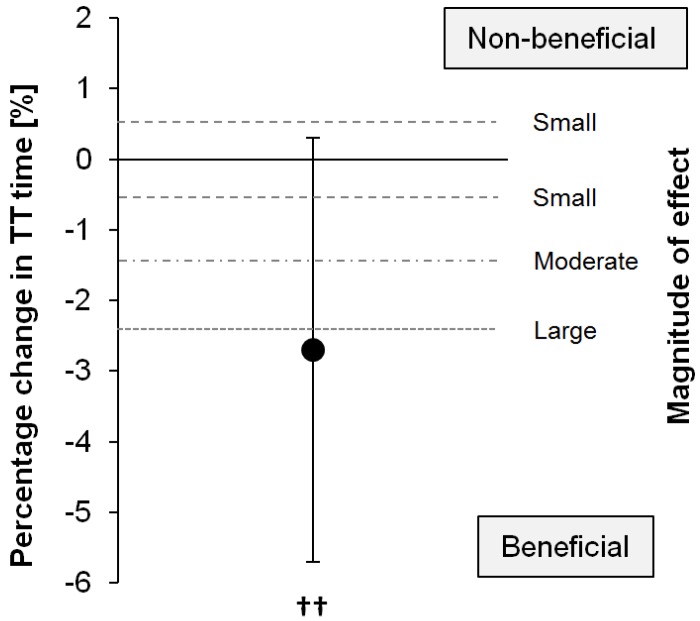
Percentage change and 90% confidence interval (CI) of cycling time trial finishing time between the Palatinose™ (PSE) and maltodextrin (MDX) trial (*N* = 20). Thresholds for small, moderate, and large effects are indicated by dashed lines. ^††^ denotes a likely small to moderate benefit, i.e., faster time trial performance with PSE compared with MDX.

The time to complete the time trial was 31.08 ± 6.27 min for the MDX and 30.05 ± 4.70 min for the PSE trial (mean change: −2.7%, 90% CI: ±3.0%; *p* = 0.147), corresponding to a likely small (89% likelihood) to moderate (77% likelihood) benefit of Palatinose™ (Figure 1).

**Figure 2:**
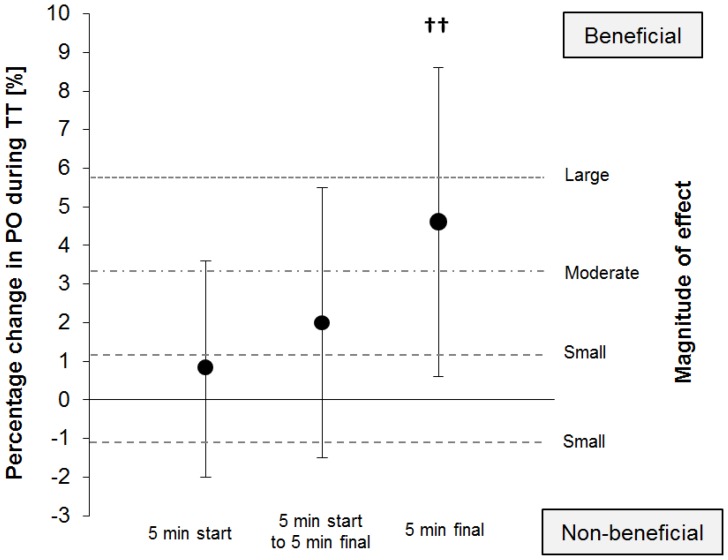
Percentage change and 90% CI of power output between the PSE and MDX trials during the first 5 min, the final 5 min, and the intermediate period of the time trial (*N* = 20). Thresholds for small, moderate, and large effects are indicated by dashed lines. ^††^ denotes a likely small benefit, i.e., higher power output with PSE compared with MDX.

The power output was consistently higher with PSE vs. MDX (Figure 2), yet a clinically relevant benefit was detected only during the final 5 min of the time trial (290.61 ± 45.85 W vs. 279.42 ± 55.91 W). The higher power output with PSE corresponds to a mean change of +0.8% (90% CI: ±2.8%, *p* = 0.608) and +2.0% (90% CI: ±3.5%, *p* = 0.327) during the first 5 min and until the final 5 min of the time trial, respectively. When the final 5 min of the time trial were analyzed, power output with PSE, compared with MDX, was higher by +4.6% (90% CI: ±4.0%; *p* = 0.053), which corresponds to a likely small benefit of Palatinose™ (93% likelihood).

Heart rate was similar between trials (time × trial interaction: *p* = 0.961), and no differences between the PSE and MDX trials, neither during rest nor during the endurance exercise, were detected (Table 1).

**Figure 3:**
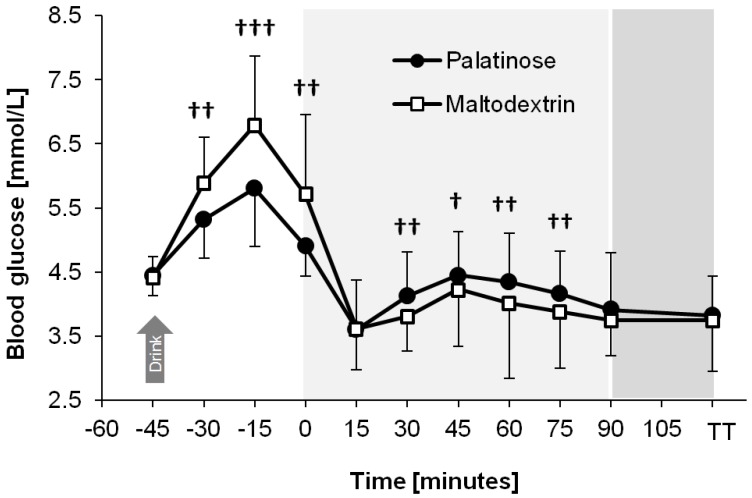
Plasma glucose profiles during the PSE and MDX trials, presented as mean ± SD (*N* = 20). The symbols ^†^, ^††^, and ^†††^ denote a possible, likely, and very likely difference between the PSE and MDX trials at the corresponding time point, respectively. From 0 min to 90 min: endurance exercise. TT = blood sample drawn upon completion of the time trial.

Figure 3 shows that the blood glucose profile differed between trials (time × trial interaction: *p* = 0.013). At rest, i.e., 30 min, 15 min, and 0 min prior to the start of exercise, consumption of PSE resulted in lower blood glucose concentrations compared with the ingestion of MDX (95% to 99% likelihood, ES = −0.61 to −0.87, moderate effect; *p* < 0.05 for all time points). After 30 min, 45 min, 60 min, and 75 min of endurance exercise, blood glucose concentrations were higher with PSE compared with MDX (63% to 86% likelihood, ES = 0.29 to 0.52, small effect; *p* > 0.05 for all time points). The reduction in glycemia following the onset of exercise was attenuated with PSE vs. MDX (93% likelihood, ES = 0.64, moderate effect; *p* = 0.034).

**Figure 4:**
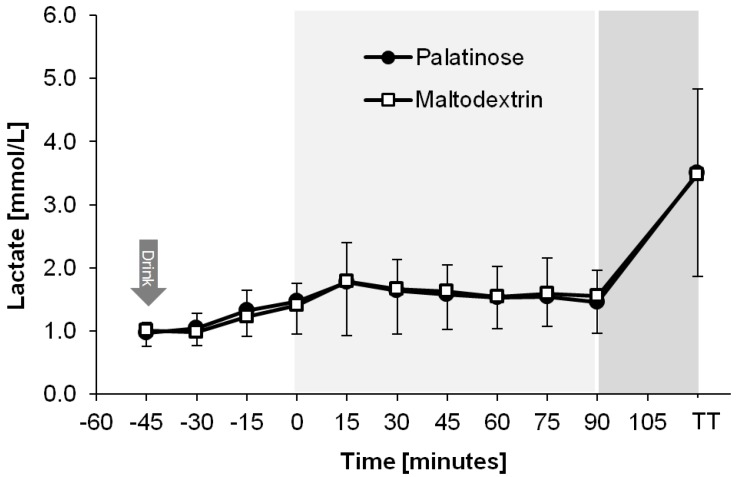
Plasma lactate profiles during the PSE and MDX trials, presented as mean ± SD (*N* = 20). From 0 min to 90 min: endurance exercise. TT = blood sample drawn upon completion of the time trial.

Figure 4 demonstrates that plasma lactate concentrations were similar between trials (time × trial interaction: *p* = 0.562), and no differences could be detected.

**Figure 5:**
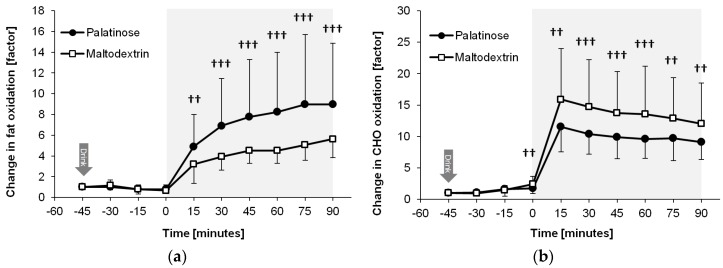
Changes in (**a**) fat oxidation and (**b**) carbohydrate (CHO) oxidation during the PSE and MDX trials, presented as mean ± SD (*N* = 17). The symbols ^††^ and ^†††^ denote a likely and very likely difference between the PSE and MDX trials at the corresponding time point, respectively. From 0 min to 90 min: endurance exercise.

Figure 5 shows that the exercise-induced increase in fat and carbohydrate oxidation differed between the MDX and PSE trial (time × trial interactions: *p* = 0.005 and *p* = 0.002). During the endurance exercise, PSE resulted in higher fat oxidation (88% to 99% likelihood, ES = 0.65 to 1.60, moderate to large effect; *p* < 0.05 for all time points from 30 to 90 min) and lower carbohydrate oxidation compared with MDX (85% to 96% likelihood, ES = −0.44 to −0.63, small to moderate effect; *p* < 0.05 for all time points from 0 to 75 min and *p* = 0.069 at 90 min). Total energy expenditure was similar between trials (1306 kcal vs. 1316 kcal for the PSE and MDX trials until 90 min, respectively; *p* = 0.579).

During the entire examination period, no adverse or unintended effects were observed. None of the subjects complained about bad palatability or gastrointestinal discomfort following ingestion of either test beverage.

## 4. Discussion

The most important finding in the present study was that cycling time trial finishing time was 30.05 ± 4.70 min following pre-exercise ingestion of low glycemic Palatinose™ and 31.08 ± 6.27 min when an isocaloric high glycemic maltodextrin beverage was consumed. The effect size of 1 min is impressive, even though under this study design the difference achieved did not reach the level of statistical significance. Using the probabilistic magnitude-based inference approach as recommended for studies in sports medicine and exercise sciences, this effect corresponds to a likely benefit of Palatinose™. This was also accompanied by a likely benefit of Palatinose™ for power output during the final 5 min of the time trial. Furthermore, pre-exercise ingestion of a low glycemic Palatinose™ beverage increased fat oxidation while reducing carbohydrate oxidation during the endurance exercise, as compared with the high glycemic maltodextrin drink. Palatinose™ ingestion also resulted in a more stable blood glucose profile with lower blood glucose increases shortly after consumption and a sustained blood glucose response during the subsequent endurance exercise protocol at 60% VO_2_max.

Official guidelines recommend that, in endurance-type sports, CHO should be the predominant source of energy within the athlete’s diet [22]. According to current guidelines, athletes should aim at increasing the amount of CHO within their diet to 6–10 g CHO/kg bodyweight/day in accordance with training duration or intensity of competition [23]; the GI of CHO is not sufficiently addressed in current guidelines.

However, the GI of ingested CHO has a distinct influence on important metabolic processes under both resting or pre-exercise conditions and during exercise. It has been previously demonstrated that the course of blood glucose and insulin levels following ingestion of low GI CHO favored a higher level of free fatty acids during exercise and was associated with enhanced fat oxidation and improved blood glucose homeostasis [6,7,24,25,26,27]. During submaximal endurance exercise, the maintenance of higher fat oxidation leads to a sparing of glycogen in muscles and particularly in the liver. It has been speculated that this glycogen sparing could lead to enhanced endurance capacity. This is the most likely theory that explains the better performance in the time trial following Palatinose™ ingestion.

An improved performance following low GI CHO ingestion could be found in some [8,10,13,26,28] but not all studies [27,29,30]. This may be due to differences in the quantity and timing of CHO ingested as well as the type, duration, and intensity of the exercise protocol. It is evident that the glycogen sparing effect of low GI CHO will not be relevant during short or high intensity exercise.

Our data show that pre-exercise ingestion of Palatinose™ was associated with higher blood glucose concentrations during the 90 min endurance exercise. The drop in glycemia following the onset of exercise, i.e., the “exercise-induced rebound glycemic response”, was substantially attenuated with PSE compared with MDX. This could be explained by the lower pre-exercise glucose and insulin response following Palatinose™ ingestion, the slow release kinetic of the monosaccharides glucose and fructose contributing to a more constant energy supply, and, of course, the higher amount of fat oxidized during the PSE trial [6,24,25].

Apart from the energy provision by CHO during exercise, there is also evidence that higher glucose concentrations improve mental performance. It has been shown that ingestion of slowly available, low GI Palatinose™ improves mental performance compared with higher GI CHO [31]. In addition, it has been demonstrated that also relatively short and highly intense exercise may be improved by pre-exercise CHO ingestion [32,33]. Therefore, it could be speculated that the higher glucose levels during the 90-min endurance exercise improved cognitive performance and reduced mental fatigue, thereby further improving stamina during the time trial [32].

Nevertheless, the study has several limitations: The number of subjects is relatively small, and some variables such as ratings of perceived exertion, muscle glycogen content, and the rate of appearance and disappearance of glucose or hormones such as cortisol have not been measured. In addition, while parameters such as blood glucose, fat, and carbohydrate oxidation were beneficial in both the magnitude-based inference approach and the conventional ANOVA, the improvement of time trial performance and power output by Palatinose™ was only beneficial in the magnitude-based inference approach and did not reach significance in the conventional statistical analysis (*p* = 0.147 and *p* = 0.053, respectively). However, we feel that the magnitude-based inference approach is well suited to determine whether a performance-enhancing effect is truly beneficial for athletes or simply an effect that is statistically significant but without any practical value in training or competition.

## 5. Conclusions

The results of the present study have shown that cycling performance was improved following pre-exercise ingestion of a low GI Palatinose™ beverage (750 mL, 10% *w*/*v*) compared with an isocaloric high GI maltodextrin drink. In the preceding 90 min endurance exercise at 60% VO_2_max, Palatinose™ resulted in a sustained blood glucose response while maintaining a higher rate of fat oxidation compared with maltodextrin, thereby reducing the reliance on CHO oxidation. These differences may have contributed to the better performance indices in the time trial test.

## Figures and Tables

**Table 1:** Average heart rate at rest and during endurance exercise for the PSE and MDX trials, presented as mean ± SD (*N* = 20).

	Palatinose™	Maltodextrin
Heart rate at rest (beats/min)	65.7 ± 8.8	65.6 ± 9.2
Heart rate during endurance exercise (beats/min)	159.2 ± 13.9	158.7 ± 13.6

## Abbreviations

The following abbreviations are used in this manuscript:

ANOVA	analysis of variance
CHO	carbohydrate
ES	effect size
CI	confidence interval
GI	glycemic index
MDX	maltodextrin
PSE	Palatinose™
SD	standard deviation
